# Alterations in Mitochondrial Oxidative Phosphorylation System: Relationship of Complex V and Cardiac Dysfunction in Human Heart Failure

**DOI:** 10.3390/antiox13030285

**Published:** 2024-02-26

**Authors:** Isaac Giménez-Escamilla, Carlota Benedicto, Lorena Pérez-Carrillo, Marta Delgado-Arija, Irene González-Torrent, Roger Vilchez, Luis Martínez-Dolz, Manuel Portolés, Estefanía Tarazón, Esther Roselló-Lletí

**Affiliations:** 1 Clinical and Translational Research in Cardiology Unit, Health Research Institute Hospital La Fe (IIS La Fe), Avd. Fernando Abril Martorell 106, 46026 Valencia, Spain; isaac_gimenez@iislafe.es (I.G.-E.); cbenmar@posgrado.upv.es (C.B.); lorena_perezc@iislafe.es (L.P.-C.); marta_delgado@externos.iislafe.es (M.D.-A.); irene_gonzalez@iislafe.es (I.G.-T.); martinez_luidol@gva.es (L.M.-D.); portoles_man@gva.es (M.P.); 2 Center for Biomedical Research Network on Cardiovascular Diseases (CIBERCV), Avd. Monforte de Lemos 3-5, 28029 Madrid, Spain; 3 Neuromuscular and Ataxias Research Group, Health Research Institute Hospital La Fe (IIS La Fe), Avd. Fernando Abril Martorell 106, 46026 Valencia, Spain; roger_vilchez@iislafe.es; 4 Centre for Biomedical Network Research on Rare Diseases (CIBERER), Avd. Monforte de Lemos 3-5, 28029 Madrid, Spain; 5 Heart Failure and Transplantation Unit, Cardiology Department, University and Polytechnic La Fe Hospital, Avd. Fernando Abril Martorell 106, 46026 Valencia, Spain

**Keywords:** mitochondria, heart failure, ischemic and dilated cardiomyopathy, OXPHOS, complex V, cardiac remodeling

## Abstract

Heart failure (HF) is a disease related to bioenergetic mitochondrial abnormalities. However, the whole status of molecules involved in the oxidative phosphorylation system (OXPHOS) is unknown. Therefore, we analyzed the OXPHOS transcriptome of human cardiac tissue by RNA-seq analyses (mRNA *n* = 36; ncRNA *n* = 30) in HF patients (ischemic cardiomyopathy (ICM) and dilated cardiomyopathy (DCM)) and control subjects. We detected 28 altered genes in these patients, highlighting greater deregulation in ICM. Specifically, we found a general overexpression of complex V (ATP synthase) elements, among them, *ATP5I* (ICM, FC = 2.04; *p* < 0.01), *ATP5MJ* (ICM, FC = 1.33, *p* < 0.05), and *ATP5IF1* (ICM, FC = 1.81; *p* < 0.001), which presented a significant correlation with established echocardiographic parameters of cardiac remodeling and ventricular function as follows: left ventricular end-systolic (*p* < 0.01) and end-diastolic (*p* < 0.01) diameters, and ejection fraction (*p* < 0.05). We also detected an increase in ATP5IF1 protein levels (ICM, FC = 1.75; *p* < 0.01) and alterations in the microRNA expression levels of miR-208b-3p (ICM, FC = −1.44, *p* < 0.001), miR-483-3p (ICM, FC = 1.37, *p* < 0.01), regulators of *ATP5I*. Therefore, we observed the deregulation of the OXPHOS transcriptome in ICM patients, highlighting the overexpression of complex V and its relationship with cardiac remodeling and function.

## 1. Introduction

Heart failure (HF) is a complex multifactorial syndrome in which the heart is unable to satisfy the metabolic demands of the organism [1]. It is classified as a global pandemic due to its high rates of morbidity and mortality worldwide which represent a significant economic and public health burden [2]. The most frequent classification divides the origin of the disease into ischemic cardiomyopathy (ICM) and non-ischemic dilated cardiomyopathy (DCM), since they constitute approximately 80% of HF diagnosis [3]. In previous studies, we have shown that HF is associated with changes in different cellular processes. Although we described alterations in the expression of several genes and proteins common to both aetiologies (ICM and DCM), we also found different molecules to be dysregulated in each group, such as in the cardiomyocyte cytoskeletal [4], natriuretic peptides pathways [5,6], nucleocytoplasmic transport [7], cellular stress response, cardiac metabolism, and mitochondrial dysfunction [8]. Thus, it is essential to know the specific mechanisms underlying the different aetiologies to promote the understanding of pathogenesis.

To satisfy the physical demands of the heart, a powerful and steady system for providing ATP is crucial [9]. Mitochondria are the main organelle responsible for producing adenosine triphosphate (ATP), the source of energy for the cells. They can produce, through the oxidative phosphorylation system (OXPHOS) which takes place at the inner mitochondrial membrane (IMM), the majority of the ATP consumed by the heart (~95%) [10]. The OXPHOS is composed of five enzymes, namely, complex I (NADH/ubiquinone oxidoreductase), II (succinate ubiquinone oxidoreductase), III (ubiquinol cytochrome c oxidoreductase), IV (cytochrome c oxidase), and V (ATP synthase). In mammals they are all multimeric and have subunits encoded both in the nuclear genome (nDNA) and the mitochondrial genome (mtDNA), with the exception of complex II. Complex I to IV form the electron transport chain (ETC) which transfers electrons through redox reactions simultaneously with the transfer of protons across the IMM, creating an electrochemical gradient of ions that drives the synthesis of ATP through the complex V [11,12].

Evidence has shown that mitochondrial abnormalities in OXPHOS and an increase in reactive oxygen species (ROS) are closely related to HF, which might be a viable therapeutic target in HF [13], although the status of molecules involved is incompletely understood. Studies on mitochondria usually focus on a single particular molecule instead of the complete cardiac mitochondrial transcriptome [14]. Nevertheless, with the developing technologies in the multiomics fields, the identification of global and specific alterations in the mitochondrial bioenergetics are able to expand the understanding of the pathogenesis of mitochondria dysfunction in the development of HF. In fact, RNA-seq is a Next Generation Sequencing (NGS) method that provides increased sensitivity for detecting uncommon sequences and an accurate single-nucleotide resolution that can be used to discriminate between highly related sequences [15].

Despite the relevance of OXPHOS, its transcriptional profile in the two most prevalent aetiologies of HF, ICM and DCM, is unknown. Thus, we designed this study to identify the molecular status in the OXPHOS system through RNA-seq assay, as well as its relationship with cardiac remodeling and function. We observed a greater deregulation of OXPHOS in patients with ICM, highlighting that the overexpression of complex V. *ATP5I, ATP5IF1*, and *ATP5MJ*, components of the ATP synthase complex, showed a relationship with echocardiographic parameters.

## 2. Materials and Methods

### 2.1. Tissue Samples Collection

Samples of myocardial tissue were obtained from near the left ventricle’s apex of explanted human hearts of patients diagnosed with HF undergoing heart transplantation and control (CNT) donors. Specifically, 36 samples were used for mRNA-seq (CNT, *n* = 10; ICM, *n* = 13; DCM, *n* = 13), 30 samples were used for ncRNA-seq (CNT, *n* = 8; ICM, *n* = 22), and 31 samples for Western Blot analysis (CNT, *n* = 6; ICM, *n* = 25). After loss of coronary circulation, the samples were maintained in 0.9% NaCl at 4 °C for 4.4 ± 3 h and stored at −80 °C until use.

For each patient, all available information was collected: clinical history, electrocardiograms, Doppler echocardiography, hemodynamic studies, and coronary angiography. ICM diagnoses were based on the presence of prior documented episodes of acute myocardial infarction, the existence of normal contractility segments co-existing with other akinetic or dyskinetic segments shown by echocardiography, and the appearance of signs of myocardial necrosis or ischemia detected by electrocardiography. The DCM diagnoses were based on the presence of a left ventricular ejection fraction (LVEF) < 40% with a cavity dilatation measured by the left ventricular end-diastolic diameter (LVEDD) > 55 mm and evaluated by echocardiography, without history of typical angina pectoris or heart attack. 

Every patient was classified according to the New York Heart Association (NYHA) functional criteria and all were receiving medical treatment in accordance with the guidelines of the European Society of Cardiology [16]. This study was approved by the Ethics Committee (Comité Etico for Biomedical Research at the La Fe University Hospital in Valencia, Spain; Code of Protocol 2016/0320, 15 November 2016) and was carried out in accordance with the principles described in the Declaration of Helsinki [17]. Each participant gave their written informed consent to participate in the study. 

The CNT samples were obtained from non-diseased hearts that could not be transplanted owing to blood type incompatibility or to surgical reasons. The cause of death of these donors was a motor vehicle accident or a cerebrovascular event. All CNT showed a normal LVEF (≥50%).

### 2.2. RNA Extraction and Integrity

Myocardial samples were homogenized in a TissueLysser LT (Qiagen, Hilden, Germany). RNA extractions were carried out using a Quik-RNA^TM^ miniprep plus kit (Zymo Research, Irvine, CA, USA) in the mRNA-seq study (CNT, *n* = 10; ICM, *n* = 13; DCM, *n* = 13), and a PureLink™ Kit (Ambion Life Technologies, Vilnus, Lithuania) in the ncRNA-seq study (CNT, *n* = 8; ICM, *n* = 22) according to the manufacturer’s instructions. RNA was quantified using a NanoDrop1000 spectrophotometer and a Qubit 3.0 fluorimeter (Thermo Fisher Scientific, Waltham, MA, USA). The purity and integrity of the RNA samples were measured using an Agilent 2100 Bioanalyser with the RNA 6000 Nano LabChip kit (Agilent Technologies, Santa Clara, CA, USA). All samples presented a 260/280 absorbance ratio > 2.0 and RNA integrity numbers ≥ 9.

### 2.3. mRNA-Seq Analysis

Poly(A)-RNA was isolated from 25 micrograms of total RNA using the MicroPoly(A) Purist kit (Ambion Life Technologies, Austin, TX, USA). Total Poly(A) was used to generate libraries for sequencing on the SOLiD 5500XL platform, following the manufacturer’s recommendations (Ambion Life Technologies, USA). No RNA spike-in controls were used. Amplified cDNA quality was analyzed by the Bioanalyser 2100 DNA 1000 kit (Agilent Technologies, USA) and quantified using the Qubit 2.0 Fluorimeter (Invitrogen, Waltham, MA, USA). The whole transcriptome libraries were used for making SOLiD templated beads following the SOLiD Templated Bead Preparation guide. Bead quality was estimated based on workflow analysis parameters. The samples were sequenced using the 50625 paired-end protocol, generating 75 nt + 35 nt (Paired-End) + 5 nt (Barcode) sequences. Quality data were measured using software SETS parameters v3.5 (SOLiD Experimental Tracking System).

The initial whole transcriptome paired-end reads obtained from sequencing were aligned with the latest version of the human genome (version GRchr37/hg19) using the Life Technologies mapping algorithm (http://www.lifetechnologies.com/, accessed on 15 May 2020), version 1.3. The standard Bioscope parameters of version 1.3 in paired-ends and whole transcriptome analysis were used. For reverse and forward reads, the seed was the first 25 nucleotides with a maximum of 2 mismatches allowed. The aligned records were reported in BAM/SAM format [18]. We eliminated the bad quality reads (Phred score < 10) using Picard Tools software, version 1.83 (http://broadinstitute.github.io/picard/, accessed on 16 May 2020).

We deposited the mRNA-seq data in NCBI’s Gene Expression Omnibus (GEO) [19], and are accessible through GEO Series accession number GSE55296 (http://www.ncbi.nlm.nih.gov/geo/query/acc.cgi?acc=GSE55296, accessed on 15 May 2020).

### 2.4. ncRNA-Seq Analysis

We obtained the cDNA libraries following Illumina’s recommendations. In summary, 3′ and 5′ adaptors were sequentially ligated to the RNA prior to reverse transcription and cDNA generation. The cDNA was enriched using PCR to create an indexed double stranded cDNA library, and size selection was performed using a 6% polyacrylamide gel. The quantity and quality of the libraries were analyzed using a 4200 TapeStation D1000 High-Sensitivity assay (Agilent, Santa Clara, CA, USA). The cDNA libraries were pooled, and the pools were sequenced using paired-end sequencing (100 × 2) in the Illumina HiSeq 2500 sequencer. The FastQC tool (v.0.11.9) was used to carry out the quality control of the raw data. Trim_galore was applied (http://www.bioinformatics.babraham.ac.uk/projects/trim_galore/, accessed on 17 May 2020) for the adapter and quality filler of raw data. Then, the insufficient quality reads (phred score < 20) were eliminated using the Picard Tools software [20]. The HT Seq software (version 0.6.0) was used for RNAs prediction estimations [21]. The raw data for each sample were mapped against the human sequences contained in mirBase [22] using the bowtie algorithm [21]. The low-quality reads were filtered using a Q20 threshold.

### 2.5. Western Blot

In this assay, 31 samples were analyzed (CNT, *n* = 6; ICM, *n* = 25). Methods used for homogenization of samples, protein determination, polyacrylamide gel electrophoresis, and Western Blot analysis were performed as previously described by Roselló-Lletí et al. [23]. Specifically, Bis-Tris electrophoresis on 4–12% polyacrylamide gels under reducing conditions was used. The primary detection antibodies used were anti-ATP5I rabbit polyclonal antibody (1:2000 dilution, ab122241), anti-ATP5IF1 mouse monoclonal antibody (1:2000 dilution, ab223779), and anti-GAPDH mouse monoclonal antibody (1:1000 dilution, ab8245) as a loading control (all of them obtained from Abcam, Cambridge, UK).

We visualized bands using an acid phosphatase-conjugated secondary antibody and a nitroblue tetrazolium/5-bromo-4-chloro-3-indolylphosphate (NBT/BCIP, Sigma Aldrich, San Luis, MO, USA) substrate system. Finally, with an image analyzer (DNR Bio-Imagining Systems, Modi’in-Maccabim-Re’ut, Israel), the bands were digitized, analyzed, and quantified with the GelQuant Pro program (v. 12.2).

### 2.6. Tissue Processing for Electron Microscopy

Left ventricle myocardial tissue samples (size 1 mm^3^) (CNT, *n* = 6; ICM, *n* = 6; DCM, *n* = 6) were fixed in a solution of 1.5% glutaraldehyde plus 1% formaldehyde in a 0.05 M cacodylate buffer, pH 7.4, for 60 min at 4 °C. Then, samples were post-fixed in 1% OsO 4 for 60 min at 4 °C, dehydrated in ethanol and embedded in Epon 812 (Sigma Aldrich, San Luis, MO, USA). Two types of sectioning were obtained, 1.5 mm semi-thin sections were stained with toluidine 0.5%, for observation with light microscopy; and 60 nm ultra-thin sections were counter stained with 2% uranyl acetate for 20 min and 2.7% lead citrate for 3 min and mounted on nickel grids, for electron microscopy observation, using a Philips CM-100 (Amsterdam, The Netherlands).

### 2.7. Statistical Methods

Data for continuous variables were expressed as the mean ± standard deviation and for discrete variables as percentage values. The data distribution was analyzed by the Kolmogorov–Smirnov test. The Student’s *t*-test and Mann–Whitney U test were used to compare continuous variables that were normally and non-normally distributed, respectively. Fisher’s exact test was used for discrete variables. Pearson’s and Spearman’s correlation coefficients were calculated to analyze the association between variables with normal distribution and non-normal distribution, respectively.

Differential RNA expression analysis between conditions was assessed using the DESeq2 method [24] (version 3.4). We considered those RNAs with a *p* value (*p* adj) corrected by FDR  ≤  0.05 as differentially expressed to avoid identification of false positives across the differential expression data [25]. Gene predictions were estimated using the cufflinks method [26] and the expression levels were calculated using the HTSeq software, version 0.5.4p3 [27]. This method eliminated the multimapped reads, and only the unique reads were considered for gene expression estimation. The edgeR method, version 3.2.4, was applied for differential expression analysis between conditions [28]. This method relies on different normalizing processes based on in-depth global samples, CG composition, and length of genes. In the differential expression process, this method relies on a Poisson model to estimate the variance of the RNA-seq data for differential expressions. R statistic and SPSS software (version 20.0) for Windows (IBM SPSS Inc., Armonk, NY, USA) were used for statistical analyses.

## 3. Results

### 3.1. Clinical Characteristics of Patients

Myocardial tissue samples from CNT individuals and HF patients from ICM and DCM aetiology were analyzed. In Table 1, we show clinical characteristics of the patients, which had a mean age of 53 ± 9, and most of them were principally men (95%). They belonged to classes III–IV of the NYHA functional classification and significant comorbidities, including hypertension and diabetes mellitus, were identified. They presented altered values in the following echocardiographic parameters: LVEF, left ventricular end-systolic diameter (LVESD) and LVEDD. CNT individuals had a mean age of 47 ± 16 years, and 60% were male. Comorbidities and other echocardiographic data were not available for the CNT group, in accordance with the Spanish Organic Law on Data Protection 15/1999.

### 3.2. mRNA Expression of Oxidative Phosphorylation Complexes and Mitochondrial Morphology in Heart Failure Patients

We used mRNA-seq technology to perform a transcriptomic analysis on the myocardial tissue of CNT donors (*n* = 10) and HF patients, diagnosed with ICM (*n* = 13) or DCM (*n* = 13) to identify differentially expressed genes associated with HF and its two aetiologies when compared with the CNT group. A total of 109 molecules involved in OXPHOS complexes were analyzed (Table S1). The selection was made through a search in the bibliography and in several databases, specifically in Pubmed, Genenames (Gene group “Mitochondrial respiratory chain complexes”) and Toppgene (the mitochondrial respiratory chain I, II, III and IV classification and mitochondrial proton-transporting ATP synthase complex from Gene Ontology groups).

**Figure 1:**
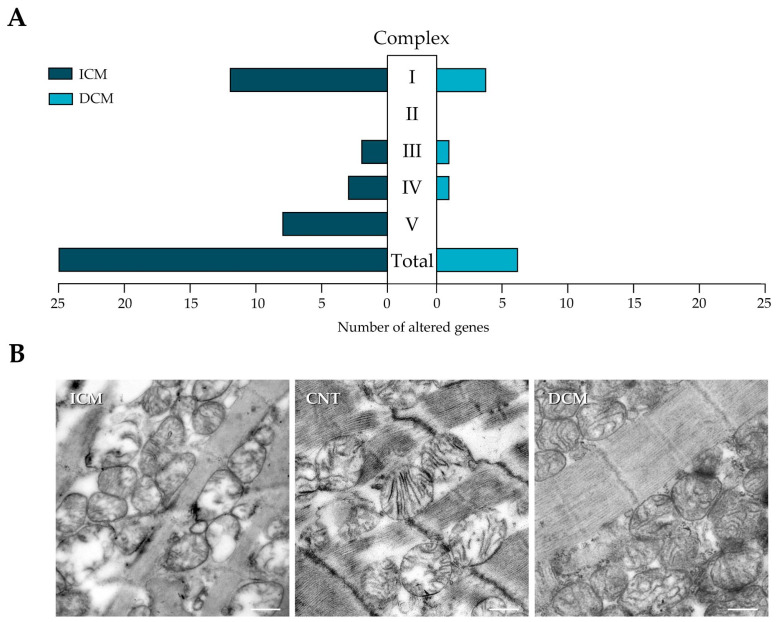
mRNA expression of oxidative phosphorylation complexes and mitochondrial morphology in heart failure patients. (**A**) Number of molecules that showed statistically significant differences in the level of expression in the oxidative phosphorylation complexes studied between control individuals and ischemic cardiomyopathy (ICM) and dilated cardiomyopathy (DCM). (**B**) Representative transmission electron microscopy micrographs of mitochondria in controls (CNT) and ICM and DCM patients. Scale bar = 500 nm.

HF patients have a general overexpression in genes of complexes I, III, IV, and V. Of all the genes analyzed, 28 were altered in these patients, and the alterations were distributed differently, with a greater presence in ICM. We found 25 altered genes in ICM patients, in contrast to the 6 altered genes in DCM, of which only 4 were coincident in both aetiologies. Most of the alterations observed in ICM patients occurred in complex I and complex V, the last one presenting a greater proportion of altered elements (36%) than complex I (24%) (Figure 1A). Representative transmission electron microscopy micrographs are shown in Figure 1B. In HF, the normal myocardial ultrastructure was disorganized showing mitochondrial fragmentation and cristae disintegration, more evident in ischaemic aetiology than in the dilated ones.

### 3.3. Complex I

The mitochondrial complex I (NADH/ubiquinone oxidoreductase) is the largest mitochondrial respiratory chain enzyme, responsible for oxidizing NADH and pumping protons. It is also considered the major source of ROS production [29]. Complex I has three functional modules named N-module (NADH binding and oxidation), Q-module (electron transfer to ubiquinone), and P-module (proton pumping) [30]. The different modules can be separated, and also, according to the types of proteins and subunits, including subunits of the α-subcomplex, β-subcomplex, iron–sulfur proteins, flavoproteins, mtDNA encoded proteins, acyl-carrier proteins, subunits C, and assembly proteins. The latter do not belong to any module.

**Figure 2:**
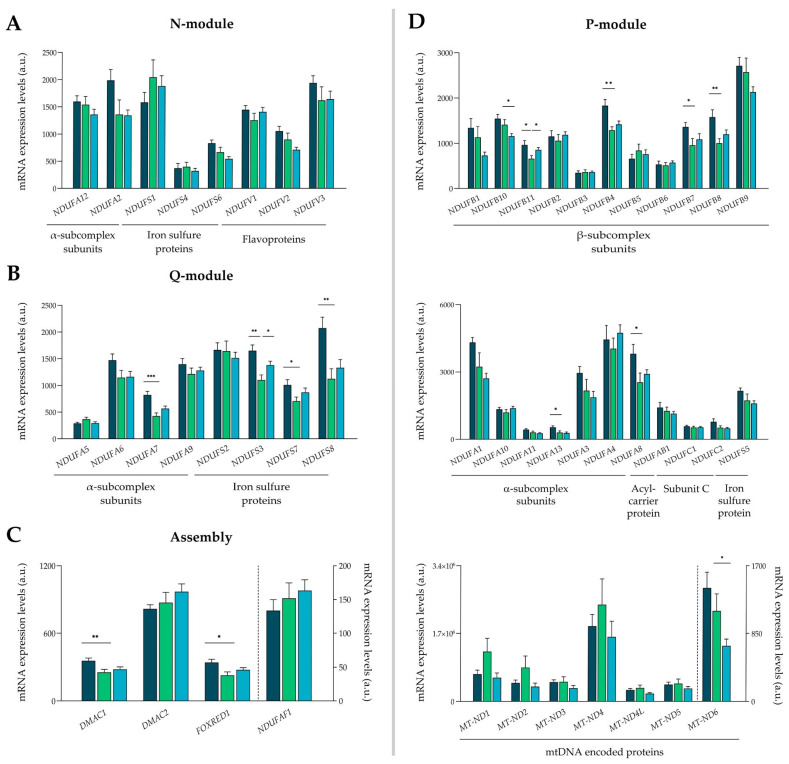
mRNA expression levels of the molecules belonging to complex I. (**A**) Subunits of N-module. (**B**) Subunits of Q-module. (**C**) Assembly proteins. (**D**) Subunits of P-module. Data are presented as the mean ± SEM; a.u., arbitrary units. Statistical differences found between ischemic cardiomyopathy (dark blue) or dilated cardiomyopathy (light blue) with control group (green): * *p* < 0.05, ** *p* < 0.01, *** *p* < 0.001.

Our data showed alterations in different subunits of the Q-module, P-module, and molecules related to the assembly of complex I, but the N-module did not present any alteration (Figure 2A). Regarding the Q-module, alterations in α-subcomplex subunits and iron–sulfur proteins were found (Figure 2B), specifically, the dysregulation in *NDUFA7* (ICM: FC = 1.92, *p* < 0.001) of the α-subcomplex subunits, and the overexpression in iron–sulfur proteins as follows: *NDUFS3* (ICM: FC = 1.50, *p* < 0.01; DCM: FC = 1.25, *p* < 0.05), *NDUFS7* (ICM: FC = 1.43, *p* < 0.05), and *NDUFS8* (ICM: FC = 1.84, *p* < 0.01). Assembly proteins that are not found within any module were overexpressed: *DMAC1* (ICM: FC = 1.40, *p* < 0.01) and *FOXRED1* (ICM: FC = 1.23, *p* < 0.05) (Figure 2C).

The P-module showed an overexpression principally in the β-subcomplex subunits (Figure 2D) as follows: *NDUFB11* (ICM: FC = 1.46, *p* < 0.05; DCM: FC = 1.30, *p* < 0.05), *NDUFB4* (ICM: FC = 1.42, *p* < 0.01), *NDUFB7* (ICM: FC = 1.42, *p* < 0.05), and *NDUFB8* (ICM: FC = 1.57, *p* < 0.01). The subunits of the α-subcomplex *NDUFA13* (ICM: FC = 1.75, *p* < 0.05) and *NDUFA8* (ICM: FC = 1.50, *p* < 0.05), belonging to the P-module, (Figure 2D) were also overexpressed. In contrast, the mitochondrial encoded gene *MT-ND6* (DCM: FC = −1.81, *p* < 0.05) was underexpressed (Figure 2D).

### 3.4. Complex II, III, and IV

**Figure 3:**
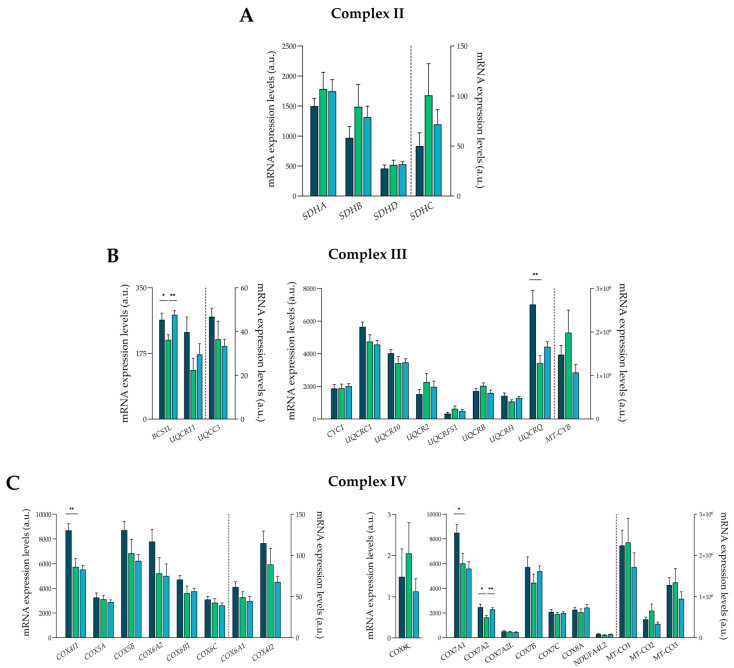
mRNA expression levels of complex II, III, and IV molecules. (**A**) Subunits of complex II. (**B**) Subunits of complex III. (**C**) Subunits of complex IV. Data are presented as the mean ± SEM; a.u., arbitrary units. Statistical differences found between ischemic cardiomyopathy (dark blue) or dilated cardiomyopathy (light blue) with control group (green): * *p* < 0.05, ** *p* < 0.01.

Complex II (succinate-ubiquinone oxidoreductase or Sdh), III (ubiquinol cytochrome c oxidoreductase) and IV (cytochrome c oxidase) belong to the ETC (Figure 3). Its main function is to carry out redox reactions that allow electrons to be transported to their final acceptor, oxygen [12].

Complex II is shared between the tricarboxylic acid cycle and the ETC and has no proton pumping activity. It comprises four subunits that are encoded by the nDNA as follows: *SDHA*, *SDHB*, *SDHD*, and *SDHC*. Our data showed that none of these subunits were altered in the studied groups (Figure 3A).

On the other hand, complex III (ubiquinol cytochrome c oxidoreductase) is responsible for accepting electrons from complexes I and II. It performs the electron transfer coupled to proton pumping, oxidizing the ubiquinol, and reducing cytochrome c [12]. We observed alterations in two of its main components, namely, the mitochondrial chaperone *BCS1L* (ICM: FC = 1.25, *p* < 0.05; DCM: FC = 1.32, *p* < 0.01) and the subunit of the catalytic core *UQCRQ* (ICM: FC = 2.05, *p* < 0.01) (Figure 3B).

Finally, the cytochrome c oxidase or complex IV performs the oxidation of cytochrome c and the reduction of oxygen to water, coupling proton translocation [12]. Different subunits were found altered in this complex: *COX4I1* (ICM: FC = 1.51, *p* < 0.01), *COX7A1* (ICM: FC = 1.41, *p* < 0.05), and *COX7A2* (ICM: FC = 1.50, *p* < 0.05; DCM: FC = 1.40, *p* < 0.01) (Figure 3C).

### 3.5. Complex V

**Figure 4:**
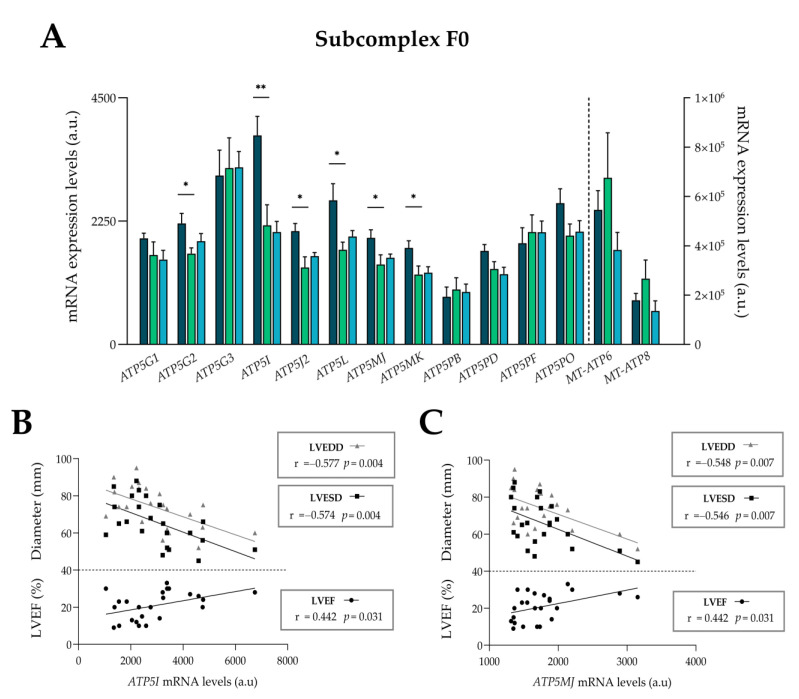

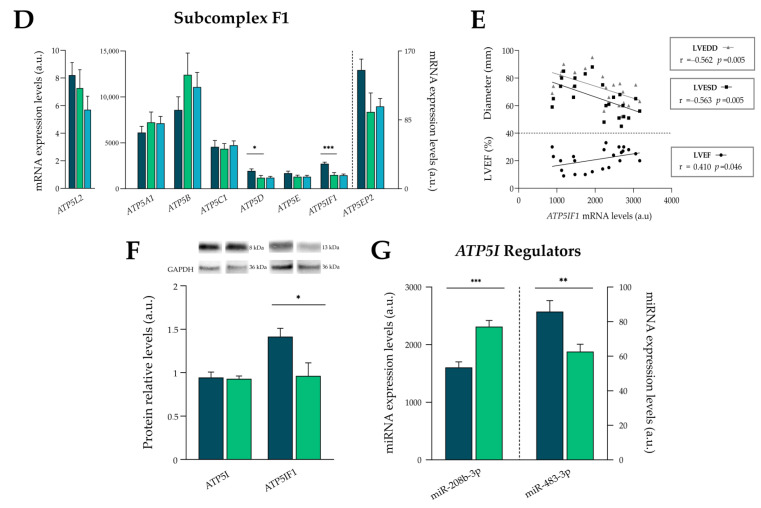
Expression levels of molecules related to complex V. (**A**) mRNA expression levels of the subunits of subcomplex F0. (**B**,**C**) Relationship between mRNA relative expression of *ATP5MJ* and *ATP5I* with the left ventricular ejection fraction (LVEF), left ventricular end-systolic diameter (LVESD), and end-diastolic diameter (LVEDD) in heart failure patients. (**D**) mRNA expression levels of the subunits of subcomplex F1. (**E**) Relationship between mRNA relative expression levels of *ATP5IF1* and LVEF, LVESD, and LVEDD in heart failure patients. (**F**) Protein expression levels of ATP5I and ATP5IF1. (**G**) miRNA expression levels whose targets are *ATP5I*. Data are presented as the mean ± SEM; a.u., arbitrary units. Statistical differences found between ischemic cardiomyopathy (dark blue) or dilated cardiomyopathy (light blue) with control group (green): * *p* < 0.05, ** *p* < 0.01, *** *p* < 0.001.

Complex V (ATP synthase) is the last complex of OXPHOS complexes. It is the enzyme that synthesizes ATP using the proton motive force produced by complexes I, III, and IV. It is divided into two topological and functional distinct regions, the membrane-intrinsic F0 and the membrane-extrinsic and matrix-facing F1 [12]. The F0 region consists of a subunit c-ring and a diverse of subunits with the function to maintain the enzyme in the IMM. The F1 portion contains five different subunits, with a triplication of the α-subunit and β-subunit [31]. All the altered subunits were overexpressed when compared to the CNT group (Figure 4). The F0 showed a greater number of altered genes as follows: *ATP5G2* (ICM; FC = 1.33, *p* < 0.05), *ATP5I* (ICM: FC = 1.76, *p* < 0.01), *ATP5J2* (ICM: FC = 1.47, *p* < 0.05), *ATP5L* (ICM: FC = 1.52, *p* < 0.05), *ATP5MJ* (ICM: FC = 1.33, *p* < 0.05), and *ATP5MK* (ICM: FC = 1.38, *p* < 0.05) (Figure 4A). In addition, the overexpression of *ATP5I* and *ATP5MJ* were inversely correlated with both diameters, LVEDD (r = −0.577 and r = −0.548, respectively; *p* < 0.01) and LVESD (r = −0.574 and r = −0.546, respectively; *p* < 0.01) and directly correlated with the LVEF (r = 0.442 and r = 0.442, respectively; *p* < 0.05) (Figure 4B, C) in HF patients. From the F1 portion, we observed the alteration of *ATP5D* or subunit delta (ICM: FC = 1.62, *p* < 0.05) and *ATP5IF1* (ICM: FC = 1.81, *p* < 0.001), inhibitor of the ATPase function of the complex V (Figure 4D). The *ATP5IF1* mRNA expression also showed an inverse correlation with both diameters, LVEDD (r = −0.562, *p* < 0.01) and LVESD (r = −0.563, *p* < 0.01), and a direct correlation with the LVEF (r = 0.410, *p* < 0.05) (Figure 4E). Protein expression levels of ATP5I and ATP5IF1 were also analyzed through Western Blot, and only ATP5IF1 showed statistically significant differences in expression between ICM patients and CNT group (FC = 1.75, *p* < 0.05) (Figure 4F). Finally, through ncRNA-seq, we estimated the expression of miRNA regulators of the expression of *ATP5IF1* and *ATP5I* (Table S2). As shown in Figure 4G, only the miRNAs whose target is ATP5I, miR-208b-3p (ICM, FC = −1.44, *p* < 0.001) and miR-483-3p (ICM, FC = 1.37, *p* < 0.01), presented alterations in their expression when compared with the ICM patients and CNT group.

## 4. Discussion

The mitochondrial alterations are closely related to the development of HF, and the bioenergetic alteration is highlighted as the principal mitochondrial abnormality associated with cardiac dysfunction [29]. Specifically, the decrease in energy production that is provided by mitochondrial oxidative metabolism leads to pathological conditions [32]. In recent decades, there has been recognition of the potential of mitochondrial dysfunction as a therapeutic target to improve cardiac function [33]. OXPHOS abnormalities are related to impaired mitochondrial ETC activity, altered ion homeostasis increased formation of ROS, aberrant mitochondrial dynamics, and shifted metabolic substrate utilization [10]. Thus, we wanted to know the global status of the main molecules involved in the OXPHOS process in mitochondrial function in HF patients, and their relationship with the cardiac function. 

Cardiomyopathies affect myocardial function and structure in a heterogeneous way, culminating in HF development. However, the different aetiologies have different clinical presentations, prognosis, and response to treatment; thus, the gene expression profiling has the potential to refine its diagnosis and prognosis [32]. Our results showed aetiology-specific alterations in patients with ICM and DCM. Specifically, patients with ICM showed deregulation of 23% of the OXPHOS genes analyzed, while patients with DCM only showed deregulation in 5.5% of them, with 3.6% shared between both aetiologies. Furthermore, and although complex I (NADH: ubiquinone oxidoreductase) is the most numerous, complex V (ATP synthase) presented the highest proportion of alterations found (36%), with generalized overexpression in patients with ICM in contrast to the non-alteration of this complex in patients with DCM. These differences in OXPHOS gene expression alterations in both aetiologies could be indicative of the differential process of mitochondrial involvement in the development of the HF.

Numerous studies have demonstrated that the failing heart exhibits anomalies in the dynamics, structure, and function of the mitochondria, resulting in a notable decrease in on-demand ATP synthesis and a notable rise in the production of harmful ROS [34]. Failure to adapt to energy stress in the heart leads to the development of mitochondrial dysfunction, which in turn feeds a maladaptive cycle that causes additional cardiac damage [10]. All complexes involved in the OXPHOS showed a general upregulation in the molecules encoded by nDNA, except complex II, which did not show any alteration. In human coronary artery disease, an increase in the expression of molecules involved in OXPHOS has been described as a compensatory mechanism for the worse mitochondrial function [35], so an overall rise in OXPHOS mRNA expression that we observed could be the result of having to produce more ATP in order to compensate for being energy depleted. In addition, we observed how this overexpression is accompanied by a disorganization in the myocardial ultrastructure, finding areas of mitochondrial fragmentation as previously described [36].

Complexes I and II (succinate:ubiquinone oxidoreductase) are the entry points for electrons to the respiratory chain and play an important role in energy metabolism due to their involvement in the regulation of ROS. Our results show a general overexpression in complex I while maintaining complex II without alteration. Previous studies have observed increased activity in complex I induced by fluoride along with a reduction in complex II [37]. These authors indicate that increased activity in complex I could lead to increased ATP and ROS production, and a reduction in complex II could suggest an attempt to reduce energy production to combat increased ROS [37]. Enhanced production of ROS can be highly detrimental and consequently damage macromolecules within mitochondria, including lipids, proteins, and mtDNA [38,39,40]. The alterations described here could be part of an adaptive mechanism of the organism to combat the deleterious effects of the increase in ROS [37].

Complex III (ubiquinol cytochrome c oxidoreductase) is the main producer of superoxide and derived ROS within the ETC [41,42,43]. We observed alterations in two molecules of this complex, *BCS1L* and *UQCRQ*. The mitochondrial chaperone BCS1L is responsible for guiding the assembly of complex III of the ETC [44]. Mutational alterations in this gene have been associated with the development of cardiac pathologies [45,46], but to date, its expression in HF is unknown. We observed the overexpression of *BCS1L* in ICM and DCM, which could indicate a common mechanism in both aetiologies. However, *UQCRQ*, encoding ubiquinol cytochrome c reductase, was only overexpressed in ICM patients. Lu et al., in mouse models, demonstrated an upregulation of UQCRQ in ICM hearts, but this alteration was not observed in DCM hearts, confirming the HF heterogeneity. Thus, UQCRQ could be a key element in the mitochondrial production of ROS in the ICM [43].

The activity of complex IV (cytochrome c oxidase) is considered a “rate-limiting step” for regulating mitochondrial ATP production since it is the terminal enzyme of the respiratory chain [47]. This complex is made up of several subunits; we observed an increase in *COX4I1*, *COX7A1*, and *COX7A2* molecules in ICM patients. In addition, *COX7A2* was also increased in DCM patients. COX7A has been described as a late-stage assembly subunit, which peripherally binds the complex allowing maturation of the final holoenzyme [48]. COX7A has two isoforms encoded by different nuclear genes: *COX7A2*, with ubiquitous expression and *COX7A1*, which is most abundantly expressed in heart and skeletal muscle. A compensatory effect of COX7A2 has been described in the absence of COX7A1. However, in cardiac tissue, both molecules appear to be necessary for the correct function of the complex [49].

Specifically, in complex V, the subunits of the F0 portion *ATP5G2*, *ATP5I, ATP5J2*, *ATP5L*, *ATP5MJ*, and *ATP5MK*, and the subunits of the F1 portion, *ATP5D* and *ATP5IF1*, were overexpressed. This general upregulation may be due to the need to generate a greater amount of ATP to match the rate of ATP consumption on a beat-to-beat basis [10,29]. In that sense, the overexpression of *ATP5I*, *ATP5MJ*, and *ATP5IF1* was significantly correlated with better cardiac function (LVEF) and measurements related with remodeling cardiac parameters (LVESD and LVEDD). We have previously observed that the protein overexpression of the alpha subunit of ATP synthase (ATP5A) correlated with a decrease in left ventricle mass, showing that the activity of ATP synthase is key in cardioprotection [50]. Here, the overexpression of ATP5IF1 at mRNA and protein level can be evidence for the prevention of ATP consumption during collapse of the electrochemical gradient, because ATP5IF1 is an inhibitor of the ATP hydrolase function. On the other hand, in ATP5I, despite being altered at the gene expression level, no protein alteration is observed. The overexpression of has-miR-483-3p, a negative regulator of ATP5I [51], could explain the absence of dysregulation at the protein level. ATP5I has been described as a partaker in the direct oxidative stress, DNA damage, responsible for ROS generation [52]. This post-transcriptional regulatory process in ATP5I expression may suggest the existence of a compensatory mechanism to maintain reduced ROS [10].

Therefore, our results show relevant alterations in the OXPHOS system, providing knowledge to understand the cellular and molecular mechanisms that contribute to poor bioenergetics of the failing heart. Of great interest is the differential behaviour of OXPHOS genes between ischemic and dilated aetiologies, in addition to the greater deregulation in patients with ICM, mainly general overexpression in complex V and the relationship with cardiac function and remodelling. Collectively, these findings highlight the promise of targeting mitochondrial dysfunction as a novel strategy to treat HF, specifically a promising and valuable tool for the differential diagnosis of DCM and ICM. Furthermore, these results open new avenues for the treatment of HF, providing numerous candidates to explore in future studies. Nowadays, therapeutic approaches aimed at restoring function of the failing heart usually target mitochondrial ROS, ion handling, substrate utilization, and mitochondrial energy production [13,53]. Therefore, knowledge of how energy production mechanisms are altered in the tissue of patients with HF may allow us to design future prospective studies of both aetiologies based on measurements of the serum expression of these molecules, targeted gene therapy models against specific molecules involved in the OXPHOS or functional studies to establish the molecular mechanisms and the clinical implications of these findings. Our results are very promising and before we can reliably apply them, more research is needed. This study has some limitations, the use of myocardial samples belonging to patients with end-stage HF is associated with wide variability between individuals and their treatment, some of which might influence the results. Even so, our study population had homogeneous aetiologies and all individuals received medical treatment in accordance with the guidelines of the European Society of Cardiology [16]. Furthermore, tissue samples were obtained from near the apex of the left ventricle; therefore, our findings cannot be generalized to all layers and regions of the left ventricle. Nonetheless, it is relevant to underline the significance of conducting this work on a sizable number of samples from explanted human hearts undergoing cardiac transplantation and control donors.

## 5. Conclusions

We showed that OXPHOS transcription is compromised in the human HF and evidenced that mRNA expression levels of the OXPHOS genes behave in a different way in the ICM and DCM aetiologies, finding greater dysregulation in ICM patients, which could indicate different mitochondrial implication in their development. These changes principally were related to a general overexpression in complex V, highlighting the relationship between the overexpression of *ATP5IF1* with the cardiac function and remodeling.

## Figures and Tables

**Table 1:** Clinical characteristics of the patients.

	mRNA-Seq		ncRNA-Seq	Western Blot
	ICM (*n* = 13)	DCM (*n* = 13)	ICM (*n* = 22)	ICM (*n* = 25)
Age (years)	54 ± 8	51 ± 11	55 ± 8	55 ± 8
Gender male (%)	100	92	100	100
NYHA class	III–IV	III–IV	III–IV	III–IV
BMI (kg/m^2^)	27 ± 4	27 ± 5	26 ± 3	27 ± 4
Hemoglobin (mg/dL)	14 ± 3	13 ± 3	14 ± 2	13 ± 3
Hematocrit (%)	41 ± 6	39 ± 7	41 ± 6	39 ± 7
Total cholesterol (mg/dL)	162 ± 41	147 ± 37	175 ± 45	192 ± 46
Prior smoking (%)	92 *	50	81	81
Prior hypertension (%)	33	17	40	48
Prior diabetes mellitus (%)	42	17	45	57
LVEF (%)	25 ± 5 *	17 ± 8	24 ± 6	23 ± 7
LVESD (mm)	57 ± 8 ***	74 ± 10	53 ± 8	54 ± 8
LVEDD (mm)	65 ± 8 ***	81 ± 8	62 ± 9	64 ± 9
Left ventricle mass index (g/m^2^)	139 ± 26 ***	245 ± 64	133 ± 30	136 ± 30
Left ventricle mass (g)	263 ± 53 ***	466 ± 105	246 ± 54	262 ± 61
Duration of disease (months)	45 ± 40	75 ± 68	36 ± 35	74 ± 67

ICM, ischemic cardiomyopathy; DCM, dilated cardiomyopathy; NYHA, New York Heart Association; BMI, body mass index; LVEF, left ventricular ejection fraction; LVESD, left ventricular end-systolic diameter; LVEDD, left ventricular end-diastolic diameter. For differences between ICM and DCM in mRNA-seq study: * *p* < 0.05; *** *p* < 0.001. Data are shown as the mean value ± standard deviation.

## Data Availability

The mRNA-seq data discussed in this publication were deposited in NCBI’s Gene Expression Omnibus [13] and are accessible through GEO Series Accession Number GSE55296 (http://www.ncbi.nlm.nih.gov/geo/query/acc.cgi?acc=GSE55296, accessed on 28 April 2014).

## Supplementary Materials

The following supporting information can be downloaded at: https://www.mdpi.com/article/10.3390/antiox13030285/s1, Table S1: Genes related to complexes of the electronic chain transport; Table S2. miRNA whose targets are *ATP5I* and *ATP5IF1*.
