# Supplementary material for: Alterations in Mitochondrial Oxidative Phosphorylation System: Relationship of Complex V and Cardiac Dysfunction in Human Heart Failure
Source: Antioxidants (Basel). 2024 Feb 26;13(3):285. doi: 10.3390/antiox13030285 (PMC10967419; doi:10.3390/antiox13030285)
Supplement: Supplementary file 1 [file antioxidants-13-00285-s001.zip › antioxidants-2885169-supplementary.pdf]

Supplementary Table 1. Genes related to complexes of the Electronic Chain Transport.

	ID	GENE	PROTEIN NAME
			Complex I - N-module
001	ENSG00000184752	*NDUFA12*	NADH dehydrogenase [ubiquinone] 1 alpha subcomplex subunit 12
002	ENSG00000131495	*NDUFA2*	NADH dehydrogenase [ubiquinone] 1 alpha subcomplex subunit 2
003	ENSG00000023228	*NDUFS1*	NADH-ubiquinone oxidoreductase 75 kDa subunit, mitochondrial
004	ENSG00000164258	*NDUFS4*	NADH dehydrogenase [ubiquinone] iron-sulfur protein 4, mitochondrial
005	ENSG00000145494	*NDUFS6*	NADH dehydrogenase [ubiquinone] iron-sulfur protein 6, mitochondrial
006	ENSG00000167792	*NDUFV1*	NADH dehydrogenase [ubiquinone] flavoprotein 1, mitochondrial
007	ENSG00000178127	*NDUFV2*	NADH dehydrogenase [ubiquinone] flavoprotein 2, mitochondrial
008	ENSG00000160194	*NDUFV3*	NADH dehydrogenase [ubiquinone] flavoprotein 3, mitochondrial
			Complex I - Q-module
009	ENSG00000128609	*NDUFA5*	NADH dehydrogenase [ubiquinone] 1 alpha subcomplex subunit 5
010	ENSG00000184983	*NDUFA6*	NADH dehydrogenase [ubiquinone] 1 alpha subcomplex subunit 6
011	ENSG00000267855	*NDUFA7*	NADH dehydrogenase [ubiquinone] 1 alpha subcomplex subunit 7
012	ENSG00000139180	*NDUFA9*	NADH dehydrogenase [ubiquinone] 1 alpha subcomplex subunit 9, mitochondrial
013	ENSG00000158864	*NDUFS2*	NADH dehydrogenase [ubiquinone] iron-sulfur protein 2, mitochondrial
014	ENSG00000213619	*NDUFS3*	NADH dehydrogenase [ubiquinone] iron-sulfur protein 3, mitochondrial
015	ENSG00000115286	*NDUFS7*	NADH dehydrogenase [ubiquinone] iron-sulfur protein 7, mitochondrial
016	ENSG00000110717	*NDUFS8*	NADH dehydrogenase [ubiquinone] iron-sulfur protein 8, mitochondrial
			Complex I - P-module: β-subcomplex subunits
017	ENSG00000183648	*NDUFB1*	NADH dehydrogenase [ubiquinone] 1 beta subcomplex subunit 1
018	ENSG00000140990	*NDUFB10*	NADH dehydrogenase [ubiquinone] 1 beta subcomplex subunit 10
019	ENSG00000147123	*NDUFB11*	NADH dehydrogenase [ubiquinone] 1 beta subcomplex subunit 11, mitochondrial
020	ENSG00000090266	*NDUFB2*	NADH dehydrogenase [ubiquinone] 1 beta subcomplex subunit 2, mitochondrial
021	ENSG00000119013	*NDUFB3*	NADH dehydrogenase [ubiquinone] 1 beta subcomplex subunit 3

022	ENSG00000065518	*NDUFB4*	NADH dehydrogenase [ubiquinone] 1 beta subcomplex subunit 4
023	ENSG00000136521	*NDUFB5*	NADH dehydrogenase [ubiquinone] 1 beta subcomplex subunit 5, mitochondrial
024	ENSG00000165264	*NDUFB6*	NADH dehydrogenase [ubiquinone] 1 beta subcomplex subunit 6
025	ENSG00000099795	*NDUFB7*	NADH dehydrogenase [ubiquinone] 1 beta subcomplex subunit 7
026	ENSG00000166136	*NDUFB8*	NADH dehydrogenase [ubiquinone] 1 beta subcomplex subunit 8, mitochondrial
027	ENSG00000147684	*NDUFB9*	NADH dehydrogenase [ubiquinone] 1 beta subcomplex subunit 9
			Complex I - P-module: α-subcomplex, acyl-carrier protein, subunit C, iron sulfure protein
028	ENSG00000125356	*NDUFA1*	NADH dehydrogenase [ubiquinone] 1 alpha subcomplex subunit 1
029	ENSG00000130414	*NDUFA10*	NADH dehydrogenase [ubiquinone] 1 alpha subcomplex subunit 10, mitochondrial
030	ENSG00000174886	*NDUFA11*	NADH dehydrogenase [ubiquinone] 1 alpha subcomplex subunit 11
031	ENSG00000186010	*NDUFA13*	NADH dehydrogenase [ubiquinone] 1 alpha subcomplex subunit 13
032	ENSG00000170906	*NDUFA3*	NADH dehydrogenase [ubiquinone] 1 alpha subcomplex subunit 3
033	ENSG00000189043	*NDUFA4*	Cytochrome c oxidase subunit NDUFA4
034	ENSG00000119421	*NDUFA8*	NADH dehydrogenase [ubiquinone] 1 alpha subcomplex subunit 8
035	ENSG00000004779	*NDUFAB1*	Acyl carrier protein, mitochondrial
036	ENSG00000109390	*NDUFC1*	NADH dehydrogenase [ubiquinone] 1 subunit C1, mitochondrial
037	ENSG00000151366	*NDUFC2*	NADH dehydrogenase [ubiquinone] 1 subunit C2
038	ENSG00000168653	*NDUFS5*	NADH dehydrogenase [ubiquinone] iron-sulfur protein 5
			Complex I - P-module: mtDNA encoded proteins
039	ENSG00000198888	*MT-ND1*	NADH-ubiquinone oxidoreductase chain 1
040	ENSG00000198763	*MT-ND2*	NADH-ubiquinone oxidoreductase chain 2
041	ENSG00000198840	*MT-ND3*	NADH-ubiquinone oxidoreductase chain 3
042	ENSG00000198886	*MT-ND4*	NADH-ubiquinone oxidoreductase chain 4
043	ENSG00000212907	*MT-ND4L*	NADH-ubiquinone oxidoreductase chain 4L
044	ENSG00000198786	*MT-ND5*	NADH-ubiquinone oxidoreductase chain 5
045	ENSG00000198695	*MT-ND6*	NADH-ubiquinone oxidoreductase chain 6
			Complex I - Assembly
046	ENSG00000137038	*DMAC1*	Distal membrane-arm assembly complex protein 1
047	ENSG00000105341	*DMAC2*	Distal membrane-arm assembly complex protein 2
048	ENSG00000110074	*FOXRED1*	FAD-dependent oxidoreductase domain-containing protein 1
049	ENSG00000137806	*NDUFAF1*	Complex I intermediate-associated protein 30, mitochondrial

050	ENSG00000140527	*WDR93*	WD repeat-containing protein 93
			Complex II
051	ENSG00000073578	*SDHA*	Succinate dehydrogenase [ubiquinone] flavoprotein subunit, mitochondrial
052	ENSG00000117118	*SDHB*	Succinate dehydrogenase [ubiquinone] iron-sulfur subunit, mitochondrial
053	ENSG00000143252	*SDHC*	Succinate dehydrogenase cytochrome b560 subunit, mitochondrial
054	ENSG00000204370	*SDHD*	Succinate dehydrogenase [ubiquinone] cytochrome b small subunit, mitochondrial
			Complex III
055	ENSG00000074582	*BCS1L*	Mitochondrial chaperone BCS1
056	ENSG00000179091	*CYC1*	Cytochrome c1, heme protein, mitochondrial
057	ENSG00000198727	*MT-CYB*	Cytochrome b
058	ENSG00000010256	*UQCRC1*	Cytochrome b-c1 complex subunit 1, mitochondrial
059	ENSG00000184076	*UQCR10*	Cytochrome b-c1 complex subunit 9
060	ENSG00000127540	*UQCR11*	Cytochrome b-c1 complex subunit 10
061	ENSG00000140740	*UQCRC2*	Cytochrome b-c1 complex subunit 2, mitochondrial
062	ENSG00000204922	*UQCC3*	Ubiquinol-cytochrome-c reductase complex assembly factor 3
063	ENSG00000156467	*UQCRB*	Cytochrome b-c1 complex subunit 7
064	ENSG00000173660	*UQCRH*	Cytochrome b-c1 complex subunit 6, mitochondrial
065	ENSG00000169021	*UQCRFS1*	Cytochrome b-c1 complex subunit Rieske, mitochondrial
066	ENSG00000164405	*UQCRQ*	Cytochrome b-c1 complex subunit 8
			Complex IV
067	ENSG00000131143	*COX4I1*	Cytochrome c oxidase subunit 4 isoform 1, mitochondrial
068	ENSG00000131055	*COX4I2*	Cytochrome c oxidase subunit 4 isoform 2, mitochondrial
069	ENSG00000178741	*COX5A*	Cytochrome c oxidase subunit 5A, mitochondrial
070	ENSG00000135940	*COX5B*	Cytochrome c oxidase subunit 5B, mitochondrial
071	ENSG00000111775	*COX6A1*	Cytochrome c oxidase subunit 6A1, mitochondrial
072	ENSG00000156885	*COX6A2*	Cytochrome c oxidase subunit 6A2, mitochondrial
073	ENSG00000126267	*COX6B1*	Cytochrome c oxidase subunit 6B1
074	ENSG00000160471	*COX6B2*	Cytochrome c oxidase subunit 6B2
075	ENSG00000164919	*COX6C*	Cytochrome c oxidase subunit 6C
076	ENSG00000161281	*COX7A1*	Cytochrome c oxidase subunit 7A1, mitochondrial
077	ENSG00000112695	*COX7A2*	Cytochrome c oxidase subunit 7A2, mitochondrial
078	ENSG00000115944	*COX7A2L*	Cytochrome c oxidase subunit 7A-related protein, mitochondrial
079	ENSG00000131174	*COX7B*	Cytochrome c oxidase subunit 7B, mitochondrial
080	ENSG00000170516	*COX7B2*	Cytochrome c oxidase subunit 7B2, mitochondrial
081	ENSG00000127184	*COX7C*	Cytochrome c oxidase subunit 7C, mitochondrial
082	ENSG00000176340	*COX8A*	Cytochrome c oxidase subunit 8A, mitochondrial
083	ENSG00000187581	*COX8C*	Cytochrome c oxidase subunit 8C, mitochondrial

084	ENSG00000185633	*NDUFA4L2*	NADH dehydrogenase [ubiquinone] 1 alpha subcomplex subunit 4-like 2
085	ENSG00000198804	*MT-CO1*	Cytochrome c oxidase subunit 1
086	ENSG00000198712	*MT-CO2*	Cytochrome c oxidase subunit 2
087	ENSG00000198938	*MT-CO3*	Cytochrome c oxidase subunit 3
			Complex V – Subcomplex F1
088	ENSG00000152234	*ATP5A1*	ATP synthase subunit alpha, mitochondrial
089	ENSG00000110955	*ATP5B*	ATP synthase subunit beta, mitochondrial
090	ENSG00000165629	*ATP5C1*	ATP synthase subunit gamma, mitochondrial
091	ENSG00000099624	*ATP5D*	ATP synthase subunit delta, mitochondrial
092	ENSG00000124172	*ATP5E*	ATP synthase subunit epsilon, mitochondrial
093	ENSG00000180389	*ATP5EP2*	ATP synthase subunit epsilon-like protein, mitochondrial
094	ENSG00000130770	*ATP5IF1*	ATPase inhibitor, mitochondrial
			Complex V – Subcomplex F0
095	ENSG00000159199	*ATP5G1*	ATP synthase membrane subunit c locus 1
096	ENSG00000135390	*ATP5G2*	ATP synthase membrane subunit c locus 2
097	ENSG00000154518	*ATP5G3*	ATP synthase membrane subunit c locus 3
098	ENSG00000169020	*ATP5I*	ATP synthase membrane subunit e
099	ENSG00000241468	*ATP5J2*	ATP synthase membrane subunit f
100	ENSG00000167283	*ATP5L*	ATP synthase membrane subunit g
101	ENSG00000156411	*ATP5MJ*	ATP synthase subunit ATP5MJ, mitochondrial
102	ENSG00000173915	*ATP5MK*	ATP synthase membrane subunit K, mitochondrial
103	ENSG00000116459	*ATP5PB*	ATP synthase F(0) complex subunit B1, mitochondrial
104	ENSG00000167863	*ATP5PD*	ATP synthase subunit d, mitochondrial
105	ENSG00000154723	*ATP5PF*	ATP synthase-coupling factor 6, mitochondrial
106	ENSG00000241837	*ATP5PO*	ATP synthase subunit O, mitochondrial
107	ENSG00000249222	*ATP5L2*	Putative ATP synthase subunit g 2, mitochondrial
108	ENSG00000198899	*MT-ATP6*	ATP synthase subunit a
109	ENSG00000228253	*MT-ATP8*	ATP synthase protein 8

WDR93, *COX6B2* and *COX7B2* expression was detected through mRNA-seq but did not have sufficient capacity to carry out its statistical study.

Supplementary Table 2. miRNA whose targets are *ATP5I* and *ATP5IF1*.

	ID	GENE	NAME
ATP5I regulators			
01	ENSG00000211991	*MIR676*	miR-676-5p
02	ENSG00000199038	*MIR210*	miR-210
03	ENSG00000207805	*MIR483*	miR-483-3p
04	ENSG00000216101	*MIR877*	miR-877-3p
05	ENSG00000215991	*MIR208B*	miR-208b-3p
ATP5IF1 regulators			
06	ENSG00000199122	*MIR148B*	miR-148b-5p
07	ENSG00000284032	*MIR29A*	miR-29a-3p
08	ENSG00000207571	*MIR615*	miR-615-3p
09	ENSG00000199053	*MIR324*	miR-324-3p
